# Recombinant Human Cytomegalovirus Expressing an Analog-Sensitive Kinase pUL97 as Novel Tool for Functional Analyses

**DOI:** 10.3390/v14102285

**Published:** 2022-10-17

**Authors:** Nadine Krämer, Martin Schütz, Uxía Gestal Mato, Lina Herhaus, Manfred Marschall, Christine Zimmermann

**Affiliations:** 1 University Medical Center, Institute for Virology, University of Mainz, 55131 Mainz, Germany; 2 Institute for Clinical and Molecular Virology, Friedrich-Alexander Universität Erlangen-Nürnberg (FAU), 91054 Erlangen, Germany; 3 School of Medicine, Institute of Biochemistry II, Goethe University, 60598 Frankfurt, Germany; 4 Research Center for Immunotherapy (FZI), University Medical Center, University of Mainz, 55131 Mainz, Germany

**Keywords:** human cytomegalovirus (HCMV), BACmid-derived recombinant HCMV, viral kinase activity, protein kinase pUL97, analog-sensitive pUL97 variant, functional analyses, pUL97-specific inhibitors

## Abstract

The human cytomegalovirus (HCMV) is a member of the beta-herpesvirus family and inflicts life-long latent infections in its hosts. HCMV has been shown to manipulate and dysregulate many cellular processes. One major interactor with the cellular host is the viral kinase pUL97. The UL97 gene is essential for viral replication, and kinase-deficient mutants of pUL97 display a severe replication defect. Recently, another group established an analog-sensitive version of the pUL97 protein. This mutant kinase can be treated with a non-hydrolysable ATP analog, thereby inhibiting its kinase function. This process is reversible by removing the ATP analog by media change. We introduced this mutant version of the pUL97 protein into the laboratory strain Ad169 of HCMV, BADwt, creating a BAD-UL97-as1 viral mutant. This mutant virus replicated normally in infected cells in the absence of the ATP analog and maintained its ability to phosphorylate its cellular substrates. However, when treated with the ATP analog, BAD-UL97-as1 displayed a defect in the production of intra- and extracellular viral DNA and in the production of viral progeny. Furthermore, in the presence of 3MB-PP1, a well-established substrate of pUL97 was no longer hyperphosphorylated. This effect was detectable as early as 4 h post treatment, which allows for studies on pUL97 without the complication of low viral titers. Nevertheless, we observed off-target effects of 3MB-PP1 on several cellular processes, which should be considered with this approach.

## 1. Introduction

The human cytomegalovirus (HCMV) is a member of the beta-herpesvirus family and shows a worldwide prevalence of 40–95% in the human population, depending on the socioeconomic, regional circumstances. Like all herpesviruses, HCMV inflicts life-long infections by establishing latency. HCMV infection is normally asymptomatic in healthy, immunocompetent individuals. However, in immunocompromised patients, such as bone-marrow recipients, HCMV reactivation can cause severe and even life-threatening complications, rendering this virus clinically highly relevant. Most importantly, HCMV represents the most frequent infection-based risk factor during pregnancy, so that congenital HCMV infection (cCMV) can cause serious and even life-threatening disease in the unborn and neonates [1].

HCMV manipulates several cellular processes in infected cells, whereby, however, many of those virus–host interactions are incompletely understood. One major viral factor of host interaction is the protein kinase pUL97, an ortholog of human cyclin-dependent kinases (CDKs) [2]. pUL97 phosphorylates many cellular and viral substrates and thus dysregulates CDK–cyclin complexes, eventually leading to a cell-cycle arrest in G1/S-phase [3]. pUL97 also serves an important role in HCMV nuclear egress, a crucial step in the viral replication cycle, by phosphorylating lamins A/C in a site-specific manner [4,5], resulting in the local distortion of the nuclear lamina during nuclear egress, [6,7,8]. Hence, kinase-depleted mutants of pUL97 display a severe replication defect, making it difficult to produce viral stock titers sufficient for experimental procedures [9,10]. 

A particularly useful tool for the study of functional aspects of kinases is the creation of so-called analog-sensitive (as) kinase versions. Basically, these mutants carry a point mutation within the ATP binding pocket of the kinase, maintaining the ability to bind ATP and maintain its kinase activity. However, the addition of a non-hydrolysable ATP-analog to the media blocks the ATP binding pocket, thereby inhibiting the phosphorylation of kinase substrates [11,12,13]. Members of Robert Kalejta’s research laboratory recently established an analog-sensitive version of the pUL97 protein, as transiently expressed using settings of plasmid transfection [14]. An important achievement of the study was the identification of a single amino acid replacement mutation within the ATP binding pocket of pUL97, which indeed, on the one hand, maintained the kinase activity of this mutant protein under normal conditions. On the other hand, however, the addition of bio-orthogonal ATP 3-methylbenzyl pyrazolopyrimidine (3MB-PP1) to the media caused a selective inhibition of pUL97 kinase activity, thereby preventing the phosphorylation of known pUL97 substrates. In the present work, we generated a recombinant HCMV expressing the analog-sensitive version of pUL97 and performed a phenotypical characterization. The use of this recombinant that expressed the pUL97 as a mutant proved to be a quick and easy way to inhibit the kinase function at any time of infection without the complication of low viral titers. In addition, the inhibition of the kinase function can be adjusted in a dose-dependent manner and is reversible when the cell media are replaced with fresh media without 3MB-PP1. Thus, we established a viral tool that enables us to study the various functions of pUL97 in the context of HCMV infection, including the topics of substrate recognition, specificity of protein binding and phosphorylation, and the in vitro efficacy of pUL97-selective inhibitory drugs. 

## 2. Materials and Methods

### 2.1. BAC-Mutagenesis and Virus Reconstitution 

To create the BAD-UL97-as1 BAC DNA, the nucleotides between 1177 and 1286 of UL97 of the parental strain Ad169/BADwt, kindly provided by Thomas Shenk [15], were replaced by a DNA fragment encoding the bacterial galactokinase galK as previously described [16]. Afterwards, the galK cassette was replaced by a DNA fragment, which encodes the sequence of UL97-as1 in which the histidin on position 411 of wild-type UL97 was replaced by a glycin by changing the coding triplet from cat to ggg. We chose the UL133-UL138-missing backbone as one part of the rationale for our studies. The deletion of the genomic region UL133-UL138 in the chosen virus context is linked to an increased dependence of the virus on pUL97 kinase activity [17,18,19,20]. For reasons of a complex regulatory interference between the viral proteins that normally are expressed by this genomic region (e.g., the early regulators of ORF-UL138 proteins and others) with the pUL97 functionality, such an HCMV strain, and mutants derived from it, are expected to be particularly sensitive to any regulatory or drug-mediated impairment of pUL97. 

For virus reconstitution BAC-clones were used to transfect HFF with BAC DNA, as described in [21]. The genomes of Ad169/BADwt and its derivatives contain a self-excisable BAC vector, leading to automatic deletion of the BAC sequence upon transfection into HFF through the expression of the CRE-recombinase [15].

### 2.2. Cell Culture

Primary human foreskin fibroblasts (HFF) were cultured in T175 flasks, containing MEM with 10% FCS, at 37 °C, 80% humidity and 5% CO_2_, as described previously [22]. Briefly, to harvest HFF, cells were washed with 1× DPBS (Sigma-Aldrich, Burlington, MA, USA, D8537) and incubated with 1× trypsin/EDTA (Gibco/Thermo Fisher Scientific, 1540054, Waltham, MA, USA) at 37 °C. After five minutes the digestion was stopped by the addition of MEM medium, cells were centrifuged at 432× *g* for five minutes and the supernatant was discarded. According to that, the cells were counted and seeded in the appropriate cell culture dishes with the corresponding cell amount. 

The following strains were used in this study: BADwt/Ad169 [15] kindly provided by Thomas Shenk, BAD-UL97-as1 was created by the exchange of the nucleotide triplet cat, coding for the amino acid histidine to ggg, coding for a glycine at position 411 in the gene of UL97 in the BADwt parental strain via homologues recombination [16].

### 2.3. The 3MB-PP1 and Maribavir Treatment

3MB-PP1 (Calbiochem Merck, 529582-5MG) and maribavir (MBV; MedChem Express, HY-16305) were added to the cell culture in a concentration of 40 µM for 3MB-PP1 or 20 µM for MBV once up to 18 h before infection or at different time points during infection. 

### 2.4. TaqMan/Kinetics

Virus titers were determined by quantitation of viral genomes in cell culture by quantitative PCR as described in [23]. The main aspects and the modifications are briefly described in the following sentences. The genome copies were estimated in infected cells as follows: 0.5 × 10^6^ cells were seeded in 10 cm dishes. On the next day, cells were infected with Ad169/BAD-wt or BAD-UL97-as1, using different virus dilutions (10 µL, 50 µL, 100 µL und 500 µL). After 6 h post-infection, the cells were harvested, adjusted to 1 × 10^6^/mL in PBS and analyzed by quantitative PCR, using an ABI 7500 Fast real-time PCR detection system. Analysis of intracellular viral DNA replication and extracellular viral DNA were performed by quantitative PCR analysis, as previously described [21]. For this, 0.5 × 10^6^ cells were seeded in 10 cm dishes and infected with 4 genomes/cell on the next day. On different days post-infection, both supernatant and cells were harvested. The supernatant was transferred into a 50 mL reaction tube, centrifuged at 2800 rpm for 10 min and stored in 1 mL aliquots at −80 °C. At 6 h post-infection and different days post-infection, the cells were washed with 1× DPBS, harvested by 1× trypsin/EDTA, centrifuged, counted and adjusted to 1 × 10^6^/mL with 1× DPBS and stored at −20 °C. The DNA was isolated both from 200 µL supernatant and 1 × 10^5^ cells by using the High Pure viral nucleic acid kit (Roche Holding AG, 11858874001) according to the manufacturer’s instructions. The quantification was performed by qPCR. 

### 2.5. Western BLOT

A total of 0.5 × 10^6^ HFF were seeded in 10 cm^2^ dishes and were either treated with 3MB-PP1, MBV or DMSO as a negative control. On the next day, the cells were infected with the respective HCMV strains with different genomes per cell (4 genomes per cell). On different days post-infection, the cells were harvested, adjusted to 1 × 10^5^ cells and lysed in 2× Laemmli sample buffer. Protein samples were separated on 10% SDS-PAGE (Life Technologies, NP0301BOX, Carlsbad, CA, USA) and transferred to PVDF membranes (Immobilon, ISEQ00010). The filters were probed with specific primary antibodies phospho-Rb (Ser807/811) (Cell Signaling, 8516S), Rb (Cell Signaling, 9309S), pp28 (kindly provided by William Britt), GAPDH (Sigma-Aldrich, G8795), pAb-UL97 (kindly provided by D.M. Coen, Harvard Medical School, Boston, MA, USA), mAb-Cyclin B1 (sc-245, Santa Cruz Biotechnology), Fluorescent-conjugated secondary antibodies (Invitrogen, A10043; Licor,926-32212) were used for detection by using the Odyssey Infrared Imager CLx (LI-COR Biotechnology, Lincoln, NE, USA). 

### 2.6. Coimmunoprecipitation Experiments (CoIP)

For the investigation of interaction patterns, HCMV-infected HFFs (in T175 flasks) were harvested and lysed 4 days post-infection (d.p.i). If indicated, cells were incubated with 40 µM of 3MB 4 h before collecting the cellular material and again during cell lysis. The coimmunoprecipitation assay (CoIP) was performed as described previously [24]. Cyclin B1 was immunoprecipitated using a pAb-cyclin B1 antibody (AF6000, R&D Systems). Immunoprecipitated samples (IP) and lysate control were subjected to standard SDS-PAGE/Wb procedures [25,26]. 

### 2.7. TMT-Based Total Proteome Analysis

The experiments were performed similar as described in [27]: cells were lysed in (2% SDS, 50 mM Tris-HCl pH 8, 150 mM NaCl, 10 mM TCEP, and 40 mM chloracetamide), heated at 95 °C for 10 min, and sonicated with Sonics Vibra-Cell. Protein lysates were precipitated by methanol/chloroform using four volumes of ice-cold methanol, one volume of chloroform, and three volumes of water. The mixture was centrifuged at 20,000× *g* for 30 min, the upper aqueous phase was removed and three volumes of ice-cold methanol added. Proteins were pelleted by centrifugation and washed twice with one volume of ice-cold methanol and air-dried. The resulting protein pellet was resuspended in 8 M urea with 10 mM EPPS pH 8.2. Protein concentration was determined with Pierce^TM^ BCA Protein Assay Kit. For digestion, 50 µg proteins were diluted to 2 M urea and incubated 1:100 with LysC and 1:100 with Trypsin overnight. The reaction was acidified using TFA (0.5%) and purified using Sep-Pakt C18 according to manufacturer’s protocol. Peptide estimation was determined with Micro BCA^TM^ Protein Assay Kit (Thermo Fischer Scientific, Waltham, MA, USA), 10 µg of peptides were TMT labeled and channels adjusted to equimolar ratios as jugged by single-injection measurements by liquid chromatography (LC)-mass spectrometry (MS). Peptides were cleaned up by C18 stage tip (washed with 3% ACN, 0.1% TFA) and fractionated according to manufacturer’s instructions using the Pierce^TM^ High pH Reversed-Phase Kit (Thermo Scientific/Thermo Fischer Scientific, Waltham, MA, USA). Samples were vacuum-dried for LC-MS measurements.

## 3. Results

### 3.1. An Analog-Sensitive Version of the Viral Kinase pUL97

**Figure 1:**
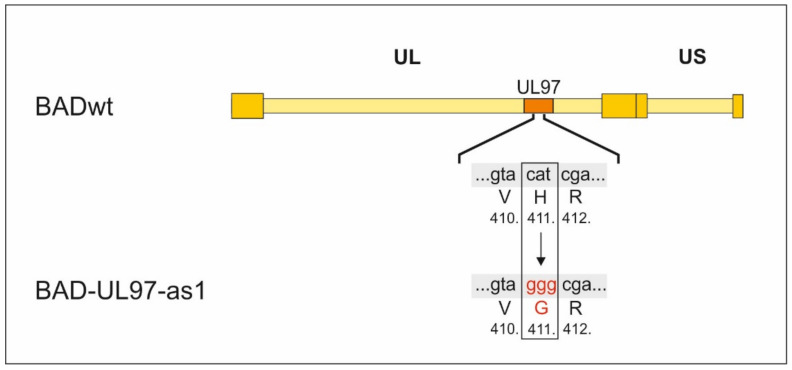
Construction of BAD-UL97-as1. The triplet cat coding for histidin on position 411 within the UL97 gene was replaced by ggg coding for glycin using the BAC recombination technique described in [16].

In order to create a viral mutant in which the kinase function of pUL97 can be reduced or inhibited in a dose-dependent manner and at a desired time point during infection, we inserted the point mutation described in [14] into the UL97 gene of the Ad169 laboratory strain of HCMV (Figure 1). For this purpose, we made use of the BAC recombination method described by others [16]. The resulting BACmid was then transfected into HFF cells to produce the viral mutant.

**Figure 2:**
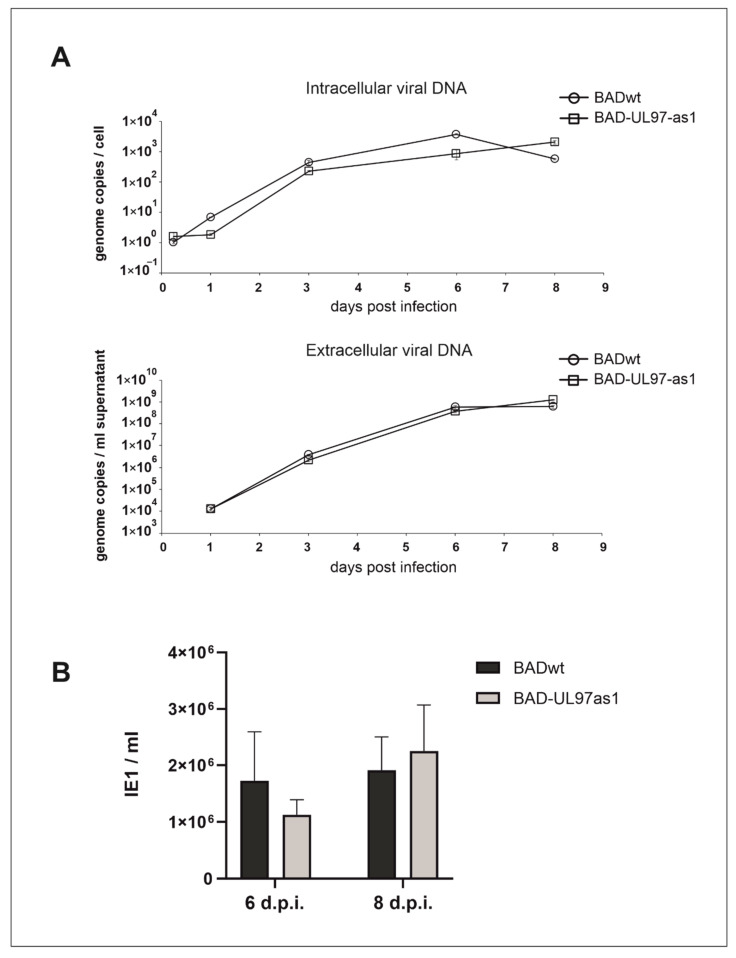
Replication kinetics of BADwt and BAD-UL97-as1. (**A**) Cells were infected with BADwt or BAD-UL97-as1 with 4 genomes/cell. Samples were taken and viral DNA was extracted from infected cells and the cell supernatant at the indicated time points. The intracellular and extracellular viral genome copies were quantified by quantitative PCR analysis. (**B**) HFF were infected with 4 genomes per cell with either BADwt or BAD-UL97-as1. After 6 and 8 days post infection, supernatants were collected and used to analyze the level of viral infectivity by serial dilution on indicator cell cultures, using staining for the immediate-early protein 1 (IE1) of HCMV at two days after inoculation. Bars represent the mean of eight technical replicates and the standard deviation is indicated by error bars.

Although analog-sensitive kinases can still bind the normal ATP molecule, a slight reduction in kinase activity of these kinases has been described in some cases [13]. To determine a possible replication defect of the UL97-as1 mutant, we performed replication kinetics of both the intracellular viral DNA, as well as the viral genomes that were released from infected human foreskin fibroblasts (HFF) (Figure 2A). Over a course of 8 days post-infection, we collected samples of UL97-as1-infected cells at 6 h, and 1, 3, 6 and 8 days post-infection (Figure 2A upper graph). Although the starting point of the UL97-as1 mutant appeared slightly delayed compared to the wild-type virus (wt), we detected no significant growth defect of the mutant. We also collected samples from the supernatants of infected cells at 1, 3, 6 and 8 days post-infection (Figure 2A lower graph) and measured the amount of viral DNA released from infected cells. We found no differences in the amount of viral DNA released from cells infected with the as1 mutant, compared to the wt virus. 

While the amount of viral DNA is an indicator for successful viral DNA replication, it is possible, that some of these genomes are not incorporated into functional viral progeny. We therefore infected HFF cell cultures with the supernatant of wild-type or mutant-infected cells, respectively. Staining for the HCMV immediate-early protein 1 (IE1) was performed two days after inoculation. There was no major difference in the amount of viral progeny released from cells infected with the BAD-UL97-as1 mutant, compared to cells infected with the parental strain (Figure 2B). 

### 3.2. Inhibition of BAD-UL97-as1 Using an ATP Analog

**Figure 3:**
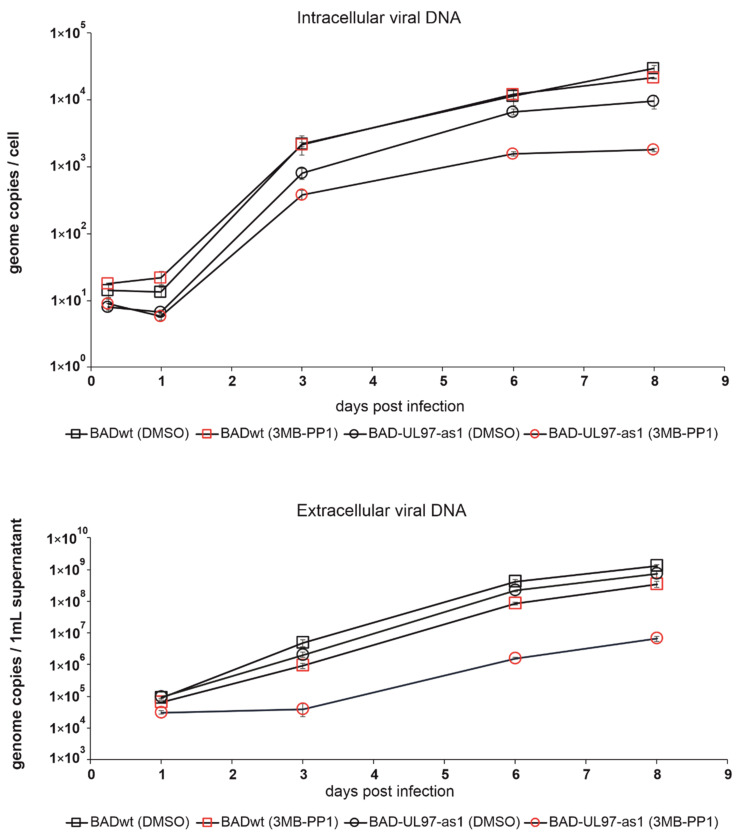
Replication kinetics of BADwt and BAD-UL97-as1 after treatment with 3MB-PP1. Cells were treated with either 40 µM of 3MB-PP1 or with DMSO one day before infection. The next day, cells were infected with BADwt or BAD-UL97-as1 with 4 genomes/cell. Samples were taken and viral DNA was extracted from infected cells and the cell supernatant at the indicated time points. The intracellular and extracellular viral genome copies were quantified by quantitative PCR analysis.

In [14], the authors tested several ATP analogs on the pUL97-as1 kinase mutant protein and found 3MB-PP1 to inhibit the kinase function most effectively. We followed that lead and chose 3MB-PP1 as the ATP-analog for further experiments. To test, whether 3MB-PP1 would indeed have an effect on the BAD-UL97-as1 mutant virus, we treated cells with 40 µM of 3MB-PP1 or DMSO the day before infection. The next day, we infected cells with either BADwt or BAD-UL97-as1. We then collected samples from infected cells at different time points and measured the amount of viral DNA present in these cells (Figure 3 upper graph). As expected, intracellular viral genomes of BAD-UL97-as1 were reduced when treated with 3MB-PP1, but not when treated with DMSO, compared to the wild type. Similarly, the amount of viral DNA released from cells infected with BAD-UL97-as1 was lower when the cells were treated with 3MB-PP1, compared to wild-type-infected cells (Figure 3 lower graph). As the literature from recent years has demonstrated, that inhibition of the pUL97 kinase activity causes viral replication defects, these results suggested, that 3MB-PP1 indeed reduced the replication of the BAD-pUL97-as1 viral strain. Interestingly, the release of viral DNA from BADwt-infected cells seemed also delayed in the presence of 3MB-PP1, even though this effect was not significant. This was surprising, because the ATP-analog was not expected to affect molecules other than the mutant kinase. However, this finding hints at off-target effects of 3MB-PP1 either on cellular processes or on other viral proteins.

### 3.3. Phosphorylation Activity of BAD-UL97-as1

**Figure 4:**
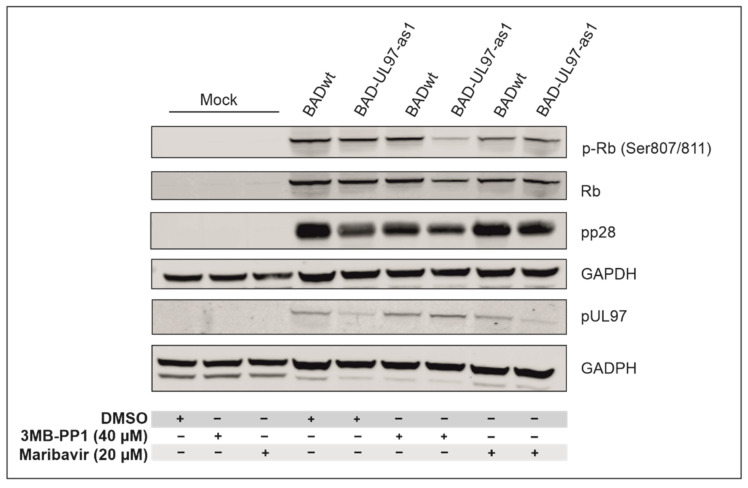
Phosphorylation of the retinoblastoma protein (Rb). Cells were pre-treated with DMSO, 3MB-PP1 or MBV one day before infection. The next day, cells were infected with BADwt or BAD-UL97-as1. Cells were harvested and lysed at 5 d.p.i. and analyzed in an SDS-PAGE and Western blot using a phospho-specific antibody for residues 807/811 of Rb. Antibodies against full protein Rb, the viral proteins pp28 and pUL97 were probed for reference.

The cellular retinoblastoma protein (Rb) is a confirmed target of pUL97. pUL97 phosphorylates Rb on Serines 807 and 811 [2,28]. In the next step, we monitored the phosphorylation status of Rb in cells infected with either BADwt or the BAD-UL97-as1 mutant, using a specific antibody for these residues (Figure 4). Cells were treated with 40 µM 3MB-PP1 or with DMSO the day before infection and samples were collected at 5 days post-infection (d.p.i.). As a positive control, we also treated cells with 20 µM of a known inhibitor of pUL97, maribavir (MBV), Livtencity^TM^ [29] and analyzed the samples using SDS-PAGE and Western blot. Uninfected (mock) cells were also treated as control.

As expected, the phosphorylation levels of Serines 807/811 of Rb increased when infected with either BADwt or BAD-UL97-as1 in the absence of 3MB-PP1 and MBV, compared to mock-infected cells. However, when BAD-UL97-as1-infected cells were treated with 3MB-PP1, the phosphorylation of Rb residues 807/811 hardly increased and instead, remained close to mock levels (Figure 4). The protein levels of pUL97 were comparable in both 3MB-PP1-treated samples, excluding the possibility that the reduction in substrate phosphorylation might result from lower pUL97-as1 protein levels. This indicates, that 3MB-PP1 indeed blocks the kinase activity of pUL97-as1 during infection. As expected, MBV reduced the levels of Rb phosphorylation in both BADwt and BAD-UL97-as1-infected cells. 

### 3.4. Incubation Duration and Off-Target Effects of 3MB-PP1

While an analog-sensitive version of pUL97 is a promising tool to study the function of this kinase in an infection background, the replication defect and resulting low viral titers of BAD-UL97-as1 during long-term treatment with the analog might cause problems in various experimental procedures. To this end, we determined the minimal period of 3MB-PP1 treatment that ensures a substantial reduction in pUL97 kinase activity without reducing the viral replication and intracellular load. For this, we infected cells with either BADwt or BAD-UL97-as1 and treated with 3MB-PP1 for either 5, 2 or 1 days or for 4 h before harvesting the cells at 5 d.p.i. Cell lysates were then run in SDS-PAGE and Western blot and analyzed using the Ser807/811-specific antibody of Rb. To monitor the viral load, we also probed for a viral protein pp28, indicating the onset of viral replication in these cells. 

**Figure 5:**
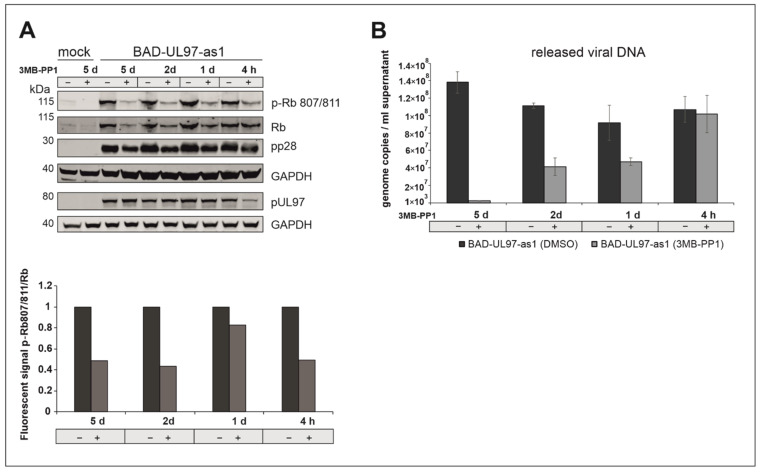
(**A**) BAD-UL97-as1 infected cells (10 genomes/cell) were harvested at 5 d.p.i. 40 µM of 3MB-PP1 was added at either 5 days, 2 days, 1 day or 4 h before harvest. The samples were analyzed by SDS-Page and Western blot analysis, using a specific antibody against the phosphosite 807/811 of Rb. The quantification was performed by measuring the protein intensity of pRb and Rb using Image Studio Lite Version 5.2.5. The ratios (pRb807/811/Rb) in dependence of 3MB-PP1 are shown. Thereby the corresponding DMSO sample was indicated as 1 (n = 2). (**B**) BAD-UL97-as1 (2 genomes/cell) infected HFFs were treated with 3MB-PP1 at different time points during infection. At 5 days, 2 days, 1 day or 4 h before harvest, the inhibitor was added. After 5 days post-infection, the supernatant was collected, DNA was isolated and the viral DNA of three technical replicates was quantified by TaqMan-PCR. The means ± standard deviations of each sample are shown.

We observed a reduction of the phosphorylation of Ser807/811 of Rb as soon as 4 h post-treatment with 3MB-PP1 (Figure 5). A treatment of 2 or even 5 days resulted in a reduction in the Rb full protein as well as in a reduction in the viral protein pp28 (Figure 5A). Furthermore, viral titers in the supernatant of BAD-UL97-as1-infected cells were reduced when treated with 3MB-PP1 for one day or longer, while viral titers remained comparable to untreated samples when only treated for 4 h (Figure 5B). 

**Figure 6:**
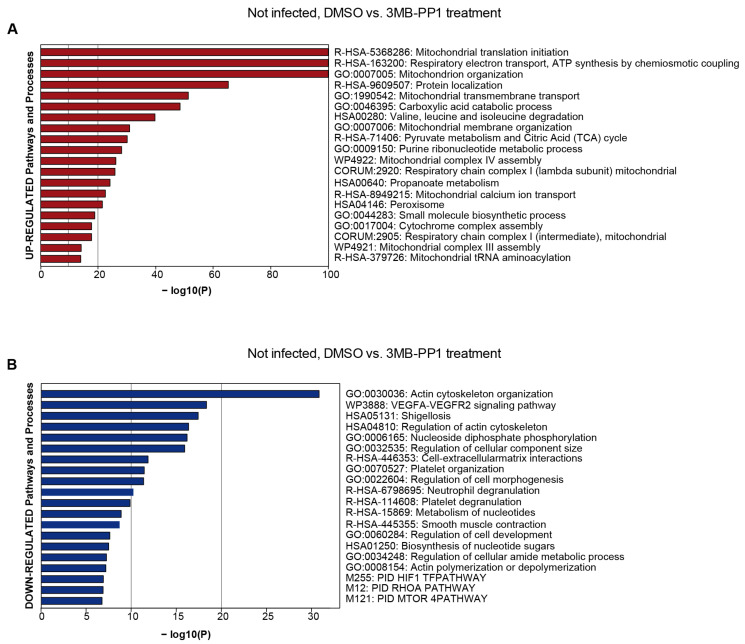
Dysregulated protein groups after 3MB-PP1 treatment in uninfected HFF. Pathway and process enrichment analysis of proteins up-regulated after 3MB-PP1 treatment (log2 ≥ 0.7 and *p*-value ≤ 0.05) (**A**). The top 20 clusters with their representative enriched terms are plotted according to their *p*-value in log base 10 (Log10 (P)). Enrichment analysis was performed with Metascape [30]. Pathway and process enrichment analysis of proteins down-regulated after 3MB-PP1 treatment (log2 ≤ −0.7 and *p*-value ≤ 0.05) (**B**). The top 20 clusters with their representative enriched terms are plotted according to their *p*-value in log base 10 (Log10 (P)). Enrichment analysis was performed with Metascape [30]. Pathway results are shown with the number of proteins found in the dataset and computed FDR for pathway enrichment (FDR < 0.001).

ATP analogs supposedly inhibit analog-sensitive kinases specifically and are not expected to interfere with other cellular processes. However, during the course of these experiments, we regularly observed an effect of 3MB-PP1 on cells infected with the wild-type virus and even uninfected cells. To exclude any severe off-target effects, we submitted lysates of uninfected cells that we had treated for 5 days with 40 µM of 3MB-PP1 to mass spectrometry to analyze possible proteome changes triggered by the ATP analog (Figure 6, Supplementary Figure S1 and Table S1). 

We observed an upregulation of cellular processes listed in Figure 6A. Many of these processes were involved in the ATP metabolism of the cell. In contrast, a number of cellular processes were downregulated, many of which intensely require ATP (Figure 6B). These findings may not be a counter-argument against the use of this tool but should be considered when analog-sensitive kinases are used. 

### 3.5. The Mutant pUL97-as1 Expressed by Recombinant HCMV Binds to Cyclins

**Figure 7:**
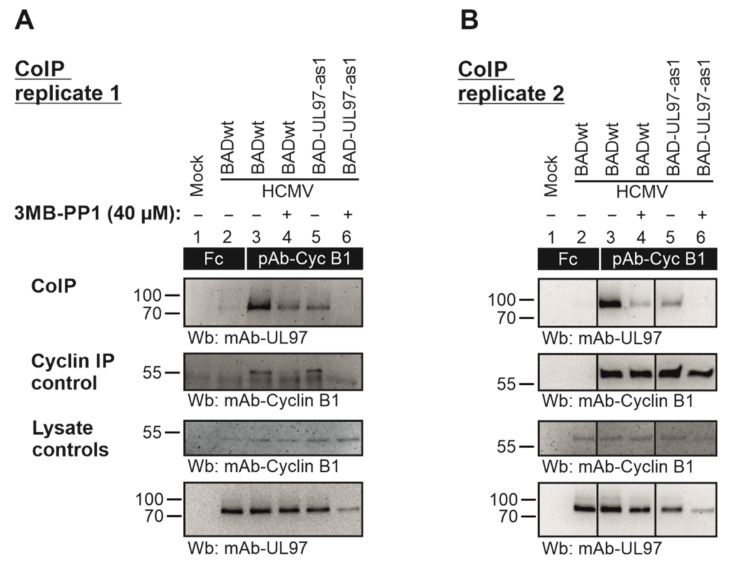
HFFs were cultivated in T175 flasks and used for infection with parental HCMV BADwt or recombinant BAD-UL97-as1 at a MOI of 0.5 for 4 d. An optional treatment with 40 µM of 3MB-PP1 (+, 3MB-PP1; −, DMSO solvent controls) was performed starting 4 h prior to sample collection and again during cell lysis. Total cell lysates were prepared and used for cyclin B1-specific CoIP with the indicated antibodies (see black boxes; Fc fragment was used as a negative control) under the continuous presence of 3MB. (**A**,**B**) show two independently produced biological replicates of this CoIP experiment, in order to illustrate the range of variability of individual signal strengths of detected protein bands. The CoIP samples were subjected to SDS-PAGE/Wb analysis (CoIP) and additional control stainings were performed to verify successful immunoprecipitation (cyclin IP control) and protein expression levels (lysate controls).

In order to address the question of whether the BAD-UL97-as1 mutant retained its ability to interact with human cyclins [31,32], a CoIP experiment was performed using BAD-UL97-as1-infected cell material in comparison to the parental wild-type pUL97 (Figure 7). Cyclin B1 was used representatively for other cyclin types since the interaction of cyclin B1 with pUL97 is uniquely dependent on pUL97 kinase activity [26]. The CoIP data indicated the interaction of cyclin B1 with pUL97-wt as described before (Figure 7A, upper panel, lane 3) [24,33]. Importantly, also the pUL97-as1 mutant clearly showed this cyclin B1 interaction (lane 5). Notably, the treatment with 3MB-PP1 led to a complete loss of cyclin B1 interaction with pUL97-as1 (lane 6), but not with wt (lane 4). Although the expression level of pUL97-as1 was found to be reduced under 3MB-PP1 treatment, we did not only note a reduced level of cyclin B1 CoIP, but found a complete loss of this signal (Figure 7, lane 6). This indicates that 3MB-PP1 treatment specifically inhibited the activity of the pUL97-as1 mutant, thus preventing cyclin B1 interaction. It should also be noted that the IP control of cyclin B1 indicated a relatively strong variation of bands. For this reason, independent replicates of this CoIP experiment were performed and clearly showed that cyclin B1 was more constant in individual other cases of this experimental setup (Figure 7B). However, we also noted a partial reduction in the pUL97-wt’s interaction with cyclin B1 (lanes 3–4) by 3MB-PP1 possibly indicating some minor effect on pUL97-wt as well. This treatment with 3MB-PP1 did not show an impairment of the expression levels of cyclin B1 and pUL97-wt, but some effect on the pUL97-as1 mutant (Figure 7A,B, lower two panels, lysate controls). As far as the detectable level of immunoprecipitated cyclin B1 was concerned, reduced signals were noted under 3MB-PP1 treatment compared to DMSO control (as possibly referring to some technical limitation of the IP efficiency caused by 3MB-PP1; Figure 7A, panel cyclin IP control, lanes 4 and 6; this was not seen in the second replicate, Figure 7B). In essence, positive signals of cyclin B1 interaction were detected for pUL97-as1 including a specific sensitivity to 3MB-PP1. Combined, the inhibition of pUL97-as1 activity by 3MB-PP1 completely blocked the detectable levels of interaction with cyclin B1 (Figure 7A,B, lanes 6). Thus, the successful cyclin B1–pUL97-as1 CoIP pointed to the preservation of a wt-like cyclin interaction phenotype of this mutant.

## 4. Discussion

Analog-sensitive kinases have been employed in various organisms for quite some time [11,12,13]. These kinases are useful tools to study any kinase and allow the inhibition of the kinase activity at any given time point in infection and in a dose-dependent manner. Additionally, this inhibition is reversible when the medium is washed off and replaced by fresh media not containing an ATP analog (Supplementary Figure S2). A possible reduction in kinase activity even without the inhibiting analog might occur in some cases but is usually not severe. In this work, we made use of a previously established analog-sensitive kinase protein of the viral kinase pUL97 of HCMV by introducing the respective mutation into the gene of UL97 in the Ad169 viral strain BADwt. 

Over a time course of 8 days, the mutant virus BAD-UL97-as1 did not display a defect of viral replication, compared to the parental strain BADwt, although the replication of intracellular viral DNA appeared slightly reduced to a non-significant amount. This indicated that the BAD-UL97-as1 virus replicates normally in the absence of 3MB-PP1.

For experiments under impaired or blocked kinase activity, we used reasonably high doses of 3MB-PP1 (40 µM), hoping to sufficiently block the kinase activity of BAD-UL97-as1. While the literature suggests lower doses of 3MB-PP1 to be sufficient, we found the best reduction in the phosphorylation of the pUL97 substrate Rb using 40 µM of 3MB-PP1 (Supplementary Figure S3). Upon infection with BAD-UL97-as1, 3MB-PP1 reduced the amounts of intracellular as well as extracellular viral DNA over a time course of 8 days, while having only little effect on titers of the parental strain BADwt. This was not surprising, because a loss of the kinase activity of pUL97 has been described previously as non-beneficial for viral replication [31,34]. 

3MB-PP1 successfully prevented the phosphorylation of the pUL97-as1 substrate Rb, as was demonstrated on the Rb residues Ser807/811. This was not observed when BAD-UL97-as1-infected cells were treated with DMSO instead, demonstrating the functionality of the mutant kinase in the absence of the inhibitor. However, a long-term treatment with 3MB-PP1 led to a replication defect of BAD-UL97-as1, as was expected. This could be a problem for several experimental procedures, when high viral titers or titers comparable to those of parental strains are required. Our findings showed, that a short-term treatment of 3MB-PP1 also led to the desired effect of inhibiting pUL97-as1 substrate phosphorylation, while having no effect on the phosphorylation activity of the wild-type kinase. Together, these results indicate a specific inhibition of the mutant viral kinase by 3MB-PP1. 

It should be mentioned that pUL97 was reported to be an active player in the HCMV-induced cellular cell cycle modulation and the induction of pseudomitosis. In this process, the main activity of pUL97, which contributes to this entire and complex phenomenon of pseudomitosis deregulation, is the massive, multisite-specific hyperphosphorylation of Rb by pUL97, leading to a G1-S phase transition of HCMV-infected cells. Our data showed that both wild-type and recombinant pUL97 were still able to perform Rb phosphorylation. However, according to the generally accepted understanding of this phenomenon, the cell cycle arrest is not a direct cause of the sole pUL97 activity, but more viral proteins appear to contribute and play additional regulatory roles [3,32,35]. Others, however, recently suggested that the reported phenotype of abnormal centrosome numbers seen in cells infected with the Ad169 strain of HCMV is rather caused by glycoprotein B (gB)-mediated cell fusion [36,37]. All together these complicated and carefully orchestrated regulatory mechanisms will require further investigation.

It should be pointed out, that 3MB-PP1 does not leave other cellular processes completely unaffected and seems to interfere with the binding of cyclins to the kinase, even in cells infected with the wild-type virus. Nevertheless, this interference does not seem to block the kinase activity or prevent target phosphorylation. In addition, a number of cellular proteins were dysregulated when cells were treated with 3MB-PP1, even in the absence of infection. Notably, ATP-producing processes were upregulated, hinting at a reaction of the cell to the presence of the non-hydrolysable ATP analog. Subsequently, processes demanding a high energy supply were decreased in the presence of 3MB-PP1. While an analog-sensitive version of the viral kinase can be a very useful tool to study the role of the kinase during infection and its manipulation of cellular targets, these findings should be considered during experimental planning.

## Data Availability

The responsible authors declare that this article fully complies with the Data Availability Statements in the section “MDPI Research Data Policies” at https://www.mdpi.com/ethics (accessed on 5 September 2022). The mass spectrometry proteomic data have been deposited in the ProteomeXchange Consortium via the PRIDE partner repository with the following dataset identifier: PXD036455.

## Supplementary Materials

The following supporting information can be downloaded at: https://www.mdpi.com/article/10.3390/v14102285/s1. Figure S1: Vulcano plot illustrating differentially abundant proteins of cells treated with 3MB-PP1 treatment. Figure S2: Reversible effect of 3MB-PP1 treatment. Figure S3: Dose-dependend effect of 3MB-PP1 on pUL97-as1.
