# Supplementary material for: Recombinant Human Cytomegalovirus Expressing an Analog-Sensitive Kinase pUL97 as Novel Tool for Functional Analyses
Source: Viruses. 2022 Oct 17;14(10):2285. doi: 10.3390/v14102285 (PMC9610083; doi:10.3390/v14102285)

Supplementary Materials:

Supplemental Figure S1. Vulcano plot illustrating differentially abundant proteins of cells treated with 3MB-PP1 treatment. The −log10 T-test P value is plotted against the log2 (fold change: 3MB-PP1/untreated). The non-axial vertical lines denote ±0.7-fold change while the non-axial horizontal line denotes P = 0.05. Protein names are listed in Supplemental Table S1.

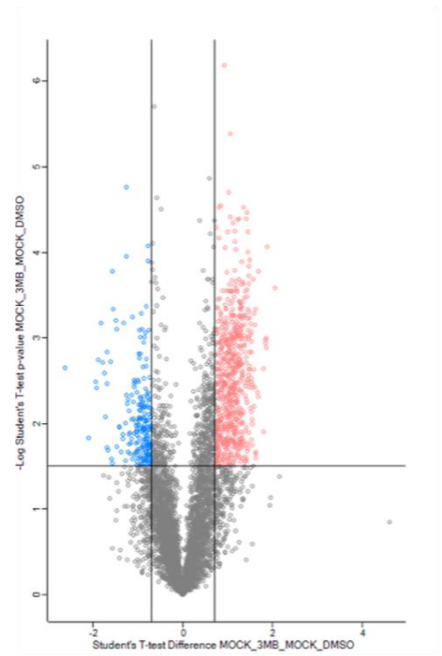

Supplemental Figure S2. Reversible effect of 3MB-PP1 treatment. HFFs were infected with the HCMV labaratory strain BAD-UL97-as1 (4 genomes/cell) and 3MB-PP1 was added at 3 days post infection. After 4h, the inhibitor was removed by exchanging the medium. Lines 4-7 represent the phosphorylation level of Rb S807/811at different time points after the removal of the inhibitor.

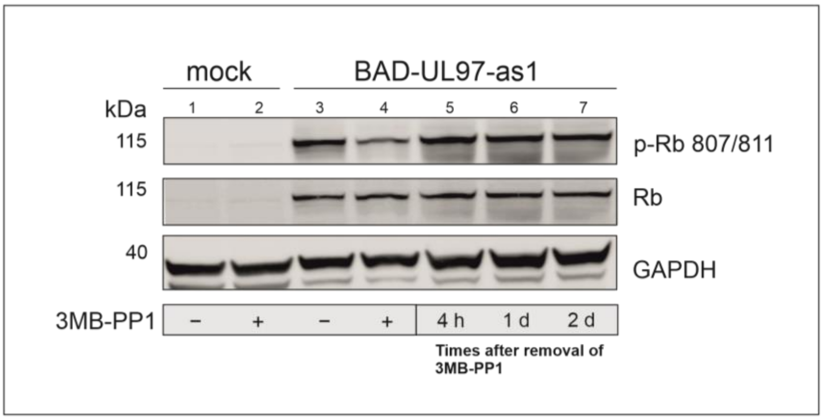

Supplemental Figure S3. Dose-dependend effect of 3MB-PP1 on pUL97-as1. HFFs were infected with the HCMV labaratory strains BADwt or BAD-UL97-as1 (4 genomes/cell) and different concentrations of 3MB-PP1 were added at the time of infection.

Cells were harvested after 5 days and phosphorylation levels of Rb were analyzed.

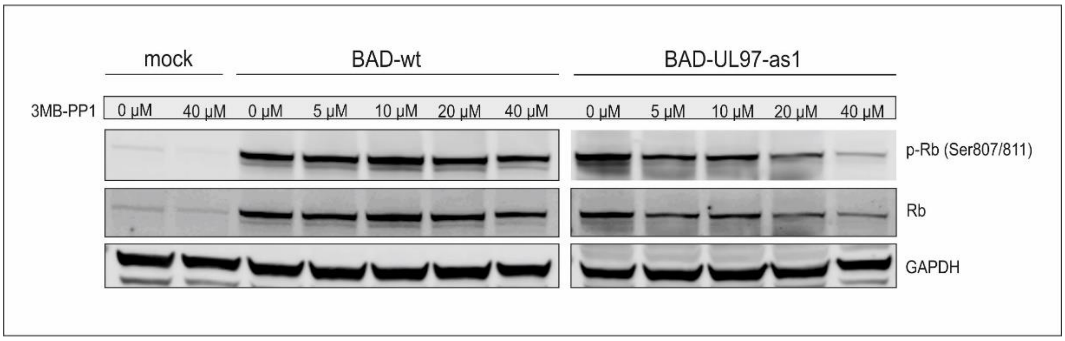

Supplement: Supplementary file 1 [file viruses-14-02285-s001.zip › viruses-1930091-supplementary.pdf]
